# Supplementary material for: Luteolin Isolated from Juncus acutus L., a Potential Remedy for Human Coronavirus 229E
Source: Molecules. 2023 May 23;28(11):4263. doi: 10.3390/molecules28114263 (PMC10254368; doi:10.3390/molecules28114263)
Supplement: Supplementary file 1 [file molecules-28-04263-s001.zip › molecules-2411361-supplementary.pdf]

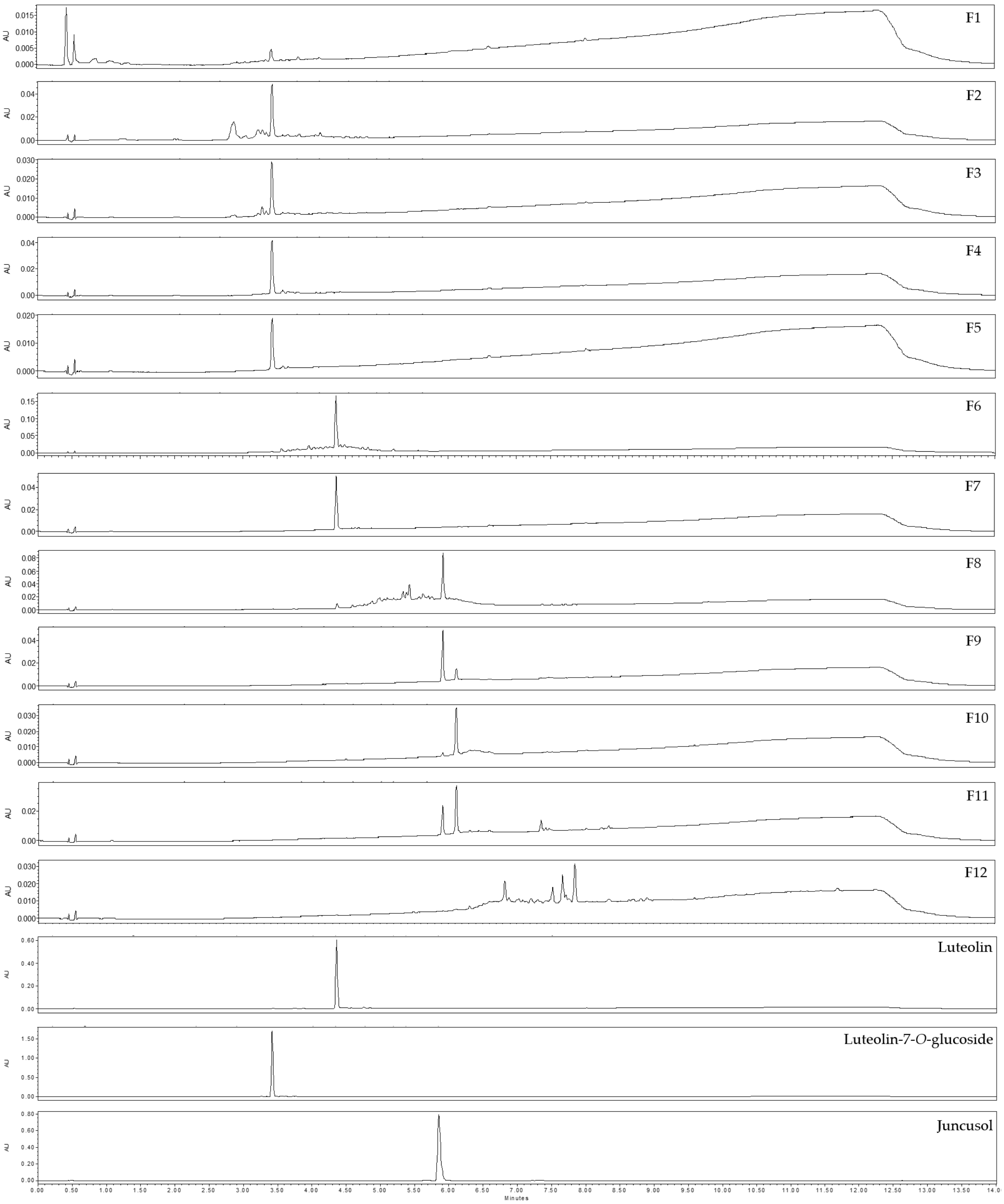


**Figure S1.** Chromatograms acquired by UHPLC-UV-MS at $\lambda$ = 254 nm of the different pre-purified fractions (F1→ F12) from the ethyl acetate sub-extract of *J. acutus* stems (JA2) and of refererence standards, luteolin, luteolin-7-O-glucoside and juncusol (at $\lambda$ = 280 nm)
